# Cortical and Spinal Excitability during and after Lengthening Contractions of the Human Plantar Flexor Muscles Performed with Maximal Voluntary Effort

**DOI:** 10.1371/journal.pone.0049907

**Published:** 2012-11-14

**Authors:** Daniel Hahn, Ben W. Hoffman, Timothy J. Carroll, Andrew G. Cresswell

**Affiliations:** 1 Department of Biomechanics in Sports, Faculty of Sport & Health Science, Technische Universität München, Munich, Germany; 2 The University of Queensland, Centre for Sensorimotor Neuroscience, School of Human Movement Studies, Brisbane, Australia; University of Alberta, Canada

## Abstract

This study was designed to investigate the sites of potential specific modulations in the neural control of lengthening and subsequent isometric maximal voluntary contractions (MVCs) versus purely isometric MVCs of the plantar flexor muscles, when there is enhanced torque during and following stretch. Ankle joint torque during maximum voluntary plantar flexion was measured by a dynamometer when subjects (n = 10) lay prone on a bench with the right ankle tightly strapped to a foot-plate. Neural control was analysed by comparing soleus motor responses to electrical nerve stimulation (M-wave, V-wave), electrical stimulation of the cervicomedullary junction (CMEP) and transcranial magnetic stimulation of the motor cortex (MEP). Enhanced torque of 17±8% and 9±8% was found during and 2.5–3 s after lengthening MVCs, respectively. Cortical and spinal responsiveness was similar to that in isometric conditions during the lengthening MVCs, as shown by unchanged MEPs, CMEPs and V-waves, suggesting that the major voluntary motor pathways are not subject to substantial inhibition. Following the lengthening MVCs, enhanced torque was accompanied by larger MEPs (p≤0.05) and a trend to greater V-waves (p≤0.1). In combination with stable CMEPs, increased MEPs suggest an increase in cortical excitability, and enlarged V-waves indicate greater motoneuronal output or increased stretch reflex excitability. The new results illustrate that neuromotor pathways are altered after lengthening MVCs suggesting that the underlying mechanisms of the enhanced torque are not purely mechanical in nature.

## Introduction

It is well known that the eccentric force of a supramaximally electrically stimulated isolated skeletal muscle or muscle fibres exceeds the isometric force at a corresponding muscle length by a factor of 1.5 to 2.4 [1]–[3]. In contrast, for human subjects performing maximum voluntary lengthening contractions (MVC), numerous studies observed no increase in eccentric torques or forces relative to an isometric contraction [4]–[8]. These findings have been attributed to a unique neural control of lengthening muscle actions [9], which is thought to be due to incomplete muscle activation caused by an inhibitory tension regulating mechanism acting during voluntary eccentric contractions [10]–[13].

On the other hand, there are a number of human studies showing that it is possible to achieve somewhat higher torques or forces during maximum voluntary lengthening contractions ranging from 1.1 up to 1.5 times of the corresponding isometric maximum [14]–[17]. Accordingly, it should be questioned why, under voluntary activation, eccentric torque or force is generally less compared to involuntary electrically stimulated lengthening contractions. If neural inhibition is the cause, then its influence may be related to increased peripheral sensory input during eccentric contractions, as suggested by Westing et al. [13], or altered motor cortical output [18], however, the exact mechanism(s) still remains elusive.

Several studies have investigated and compared neural control of lengthening and shortening contractions [19]. However, these studies cannot provide direct evidence for the underlying mechanisms of potential inhibition during maximum lengthening contractions compared to an isometric contraction, which has been examined only by few studies. Duclay & Martin [20] found reduced H-reflexes but unchanged V-waves during lengthening as compared to an isometric contraction, whereas changes in H-reflex were mainly attributed to presynaptic inhibition of Ia afferents and homosynaptic postactivation depression. In combination with unchanged V-waves it was therefore concluded that the spinal loop is specifically modulated. To identify the location (cortical vs. spinal) of specific modulations in excitability during lengthening compared to isometric MVCs, Gruber et al. [21] recorded motor evoked potentials (MEPs) as well as cervicomedullary motor evoked potentials (CMEPs) elicited in the elbow. Compared to isometric MVCs they found smaller sizes of MEPs and CMEPs during stretch, while the ratio of MEP/CMEP increased. Based on these results and the interpretation of MEPs and CMEPs [22], it was suggested that there is reduced excitability at the spinal level but enhanced motor cortical excitability during lengthening compared to isometric MVCs. This was confirmed in a further study [23] for soleus muscle, where MEPs and H-reflexes were analysed. Both responses were reduced in size during lengthening compared to isometric MVCs, which led the authors to the conclusion that the specific modulation of the corticospinal excitability during lengthening MVCs depends mainly on pre- and postsynaptic inhibitory mechanisms acting at the spinal level.

However, in all the three mentioned studies on neural control of lengthening MVCs compared to isometric MVCs [20], [21], [23], subjects failed to achieve torques during the voluntary lengthening contractions that exceeded those produced under isometric conditions. As previous literature has indicated that higher torques can be present during voluntary lengthening contractions, the results are difficult to interpret as evidence regarding putative neural inhibition. Accordingly the first aim of the current study was to investigate the sites of specific modulations in the neural control of maximal voluntary lengthening contractions of the plantar flexor muscles when there is enhanced torque during stretch [so called force enhancement (FE)] compared to purely isometric MVCs. Because stretch of muscle fascicles and higher torques during lengthening MVCs should increase Ia and Ib afferent activity, we hypothesize that potential modulations in neural control therefore depend on feedback via spinal loops.

**Figure 1:**
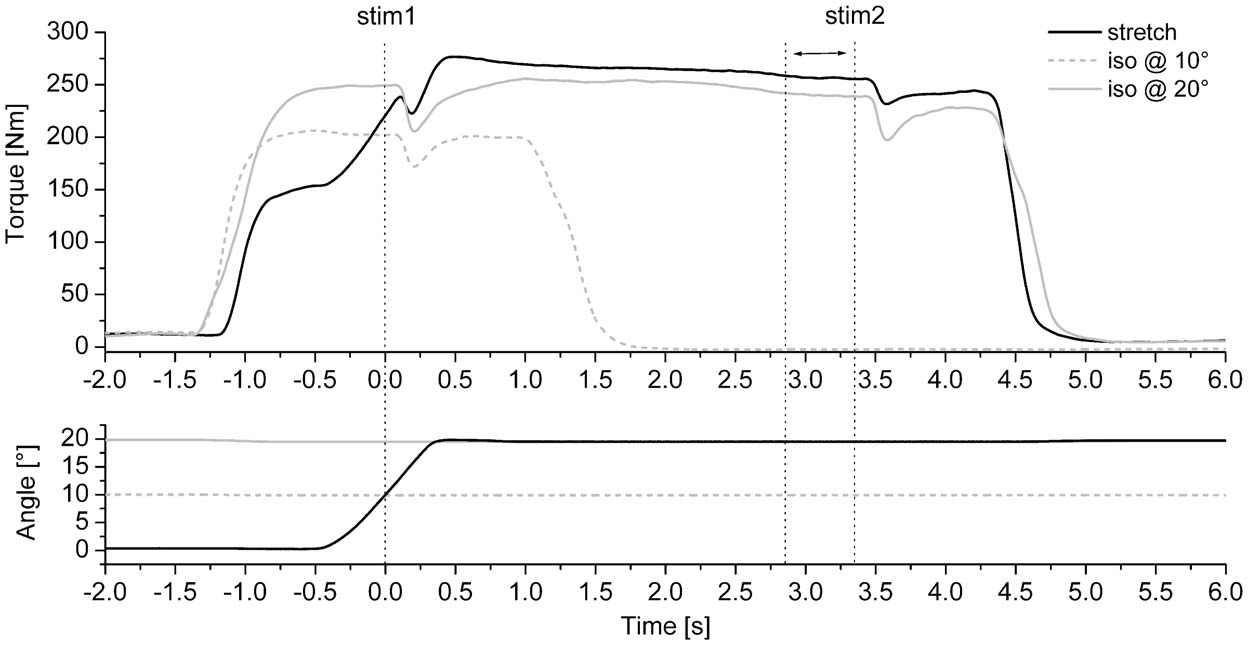
Example data of isometric reference contractions (grey lines) at 10° (dashed) and 20° (solid) dorsiflexion and an isometric-eccentric-isometric Δ20° stretch contraction (black lines). Top traces show torque while bottom traces show the corresponding ankle joint angle. The first and last dotted vertical lines indicate the times of the superimposed stimulations during and after stretch. The arrow between dotted vertical lines two and three illustrates the 500 ms window prior to stimulation 2 which was used to determine RFE. Note that there are no additional twitches after stimulation, neither during nor after stretch, indicating that voluntary activation was near-maximal.

Besides an increase in torque during lengthening MVCs compared to isometric MVCs, there is extensive evidence of a persistent increase in the steady-state isometric force following lengthening when compared to the steady-state force of a purely isometric contraction at the same muscle length as after lengthening (Fig. 1). This so called residual force enhancement (RFE) has been observed for all kind of muscle preparations ranging from single sarcomeres [24] to maximally and submaximally voluntarily contracting in vivo human muscles [14], [15], [25], [26]. Although the exact mechanism(s) remain unclear, peripheral factors underlying RFE appear to involve both passive and active components. While titin is thought to be the main passive structure contributing to RFE [27], [28], possible active mechanisms include a stretch-induced increase in the number of attached cross bridges, and/or an increase in the average cross-bridge force [24]. Another factor that has been suggested to play a role in the development of RFE are half sarcomere non-unifomities [28], [29]. Furthermore, the attachment of the second motor domain of myosin, i.e. the formation of an additional cross-bridge binding between the myosin light chain second head and actin, might contribute to higher forces after stretch [30].

In addition, little is known about neuromuscular control states following lengthening contractions. When maintaining a constant torque or force during submaximal voluntary contractions, surface EMG was found to be lower following lengthening compared to purely isometric contractions [31], [32]. Additionally, despite lower surface EMG, discharge rate of single motor units following lengthening was unaltered in comparison to a purely isometric contraction [33]. From these findings it was concluded that there might be derecruitment of units after stretch [33], and therefore saving of metabolic energy, that is a more efficient, optimized muscle activity after stretch [32].

Therefore the second aim of this study was to investigate the corticospinal excitability after eccentric lengthening contractions of the plantar flexor muscles, at a time where residual force enhancement also exists. We hypothesize that there is reduced neural drive and/or an enhancement of the effectiveness of the neuromuscular system.

## Materials and Methods

### Ethics Statement

Ten healthy subjects (29.1±6.6 yr., 1.77±0.08 m and 73.4±11.8 kg) with no history of ankle joint injury or neuromuscular disease participated in this study. Free, written informed consent was obtained and the protocol was approved by the Medical Research Ethics Committee of The University of Queensland, Australia and conducted according to the Declaration of Helsinki.

### Stimulation

Peripheral electrical nerve stimulation of the tibial nerve in the popliteal fossa was used to evoke maximal M-waves (M_max_) and V-waves in soleus. When supramaximal stimulation of a mixed nerve is superimposed on MVCs there can be a late response that is likely to be largely produced by the Ia reflex pathway. As such, it is a form of H-reflex, however, it is typically termed a V-wave [34]. That is because in contrast to typical H-reflex recordings at low intensity stimulation, during supramaximal stimulation of a mixed nerve the afferent signal is prevented from cancelation due to collision of antidromic action potential produced by the stimulation with action potentials produced via volition during MVC [35]. Cervicomedullary stimulation between the mastoids was used to activate descending spinal pathways and evoke potentials (cervicomedullary motor evoked potentials, CMEPs) in soleus and transcranial magnetic stimulation (TMS) over the cortex was used to obtain motor evoked potentials (MEPs) in the same muscle.

Electrical stimulation to the tibial nerve was delivered as a single current pulse of 1 ms duration by a constant-current stimulator (DS7AH, Digitimer Ltd, UK). Current passed from a cathode (Ag/AgCl electrode, Tyco Healthcare, Germany) placed within the popliteal fossa to an anode (coal rubber pad, 10.2×4.6 cm, Empi, USA) positioned, just proximal to the patella. The intensity used to evoke maximal M-waves of soleus muscle at rest was multiplied by a factor of 1.5 for supramaximal motor nerve stimulation during the contractions.

Cervicomedullary stimulation was delivered as a single voltage pulse of 100 µs using a constant voltage stimulator (D180A, Digitimer Ltd, UK) via electrodes (Ag/AgCl electrode, Tyco Healthcare, Germany) placed over the mastoid processes, with the cathode on the left side of the head. Since the size of CMEPs grows with contraction intensity [36] and becomes relatively less uncomfortable compared to CMEPs delivered at rest, the necessary stimulation intensity to evoke a muscle response was defined by increasing stimulator output during isometric contractions at a given torque level of 50% MVC until CMEPs were clearly visible compared to the background EMG. Submaximal contractions of 50% MVC were further chosen in order to avoid fatigue during the adjustment of stimulation intensity. During the experiment cervicomedullary stimulation was delivered when subjects performed MVCs.

TMS (Magstim 200^2^, The Magstim Company Ltd, UK) was delivered using a 12-cm double-cone coil positioned over the motor cortex, slightly left of the vertex, with monophasic current pulses running through the centre of the coil from anterior to posterior. The optimal position and intensity of stimulation were determined while subjects performed isometric plantar flexions at 50% MVC. To locate the optimal hotspot for stimulation, initially the vertex was determined through measurement of the head. The handheld coil was placed over the cortex and several stimuli were given at different locations slightly left of the vertex until the hotspot was found. The hotspot was defined as the stimulation site where soleus showed the largest MEP in response to a given supra-threshold stimulation intensity. Once determined the position of the coil was then marked on the scalp by a permanent marker and the intensity of stimulation was adjusted such that the peak-to-peak amplitudes of the MEPs in soleus were closely matched to those of the CMEPs, and that both could increase as well as decrease. This individual intensity was used during the experimental trials and the coil was maintained at its marked hotspot by the hand of an experienced experimenter.

### Experimental Procedures

Subjects attended two sessions on two different days, with at least one rest day between sessions. In the first familiarisation session, subjects were trained to perform maximal voluntary isometric and lengthening contractions. In the second session, subjects performed the required test protocol that gave the results described here. At the beginning, subjects performed a short standardised warm-up followed by the set-up of stimulation parameters for motor nerve stimulation, cervicomedullary stimulation and TMS. Then subjects performed a series of 36 MVCs with superimposed motor nerve stimulation, cervicomedullary stimulation and TMS as described below. The series of MVCs included three different contraction types: two maximal voluntary isometric reference contractions (one at 10° and the other at 20° of dorsiflexion with 0° defined as the sole of the foot being at right angles to the shank) and one maximal voluntary lengthening contraction. The latter started at 0° with a 1 s isometric period prior to the dynamometer moving to give a 20° stretch at an angular velocity of 30°s^−1^. Once at the final position (20° dorsiflexion), subjects performed an isometric contraction for 4 s. Subjects were instructed to develop their maximum plantar flexion force during the initial 1 s period and then maintain this effort for the duration of the test. During the isometric MVCs at 10° dorsiflexion only one simulation was delivered (stimulation 1), whereas during the isometric MVCs at 20° dorsiflexion and the lengthening contractions two stimulations were given (stimulation 1, stimulation 2). For lengthening contractions, stimulations were delivered as the ankle angle passed through 10° of dorsiflexion during lengthening (stimulation 1) and 3 s after the completion of the stretch during the subsequent steady-state isometric contraction with the ankle at 20° dorsiflexion (stimulation 2). The time interval between stimulation 1 and stimulation 2 was approximately 3.4 seconds. For the purely isometric contractions stimulations were delivered at the same instances in time as during the lengthening contractions in relation to the beginning of the contractions (Fig. 1). Subjects were required to perform three repetitions of each contraction type for motor nerve stimulation and cervicomedullary stimulation and six repetitions for TMS. This resulted in a total of 15 motor nerve stimulations, 15 cervicomedullary stimulations and 30 TMS superimposed on MVCs. In order to avoid fatigue a minimum rest of 3 min was enforced between all contractions that were performed in a randomised order. Strong and consistent verbal encouragement was given by the experimenters during all contractions.

### Torque and EMG Measurement

Ankle joint torque during maximal voluntary plantar flexion was measured using a dynamometer (Biodex®, System 3, USA) while subjects lay prone on the bench of the dynamometer with their right ankle strapped to the foot-plate (Fig. 1). Ankle joint axis and the rotation axis of the dynamometer were aligned carefully for all measures and the strapping was sufficiently tight to prevent heel lift from the force-plate when performing MVCs.

To analyse motor responses to stimulation and pre-stimulus muscular activity during voluntary contractions surface electromyographic activity (EMG) was recorded from soleus, medial gastrocnemius and tibialis anterior muscles. Self-adhesive electrodes (Ag/AgCl electrode, Tyco Healthcare, Germany) were placed over the muscles in a bi-polar configuration with an inter-electrode distance of approximately 20 mm, while a single ground electrode of the same type was attached to the fibular head. Skin preparation and electrode placement were done according to SENIAM [37] guidelines. EMG recordings were amplified 1000 times (NL844 and NL820A, Digitimer Ltd, UK) and band-pass filtered between 10 and 400 Hz (NL125, Digitimer Ltd, UK). All data was sampled at 5 kHz and synchronized using a 16-bit Power1401 and Spike2 data collection software (Cambridge Electronic Design, UK).

### Data Analysis

Dependent variables were plantar flexion torque, pre-stimulus EMG activity, M_max_, V-waves, CMEPs and MEPs. These parameters were analysed at two specific instances in time (stimulation 1 and stimulation 2) for all contraction types.

**Figure 2:**
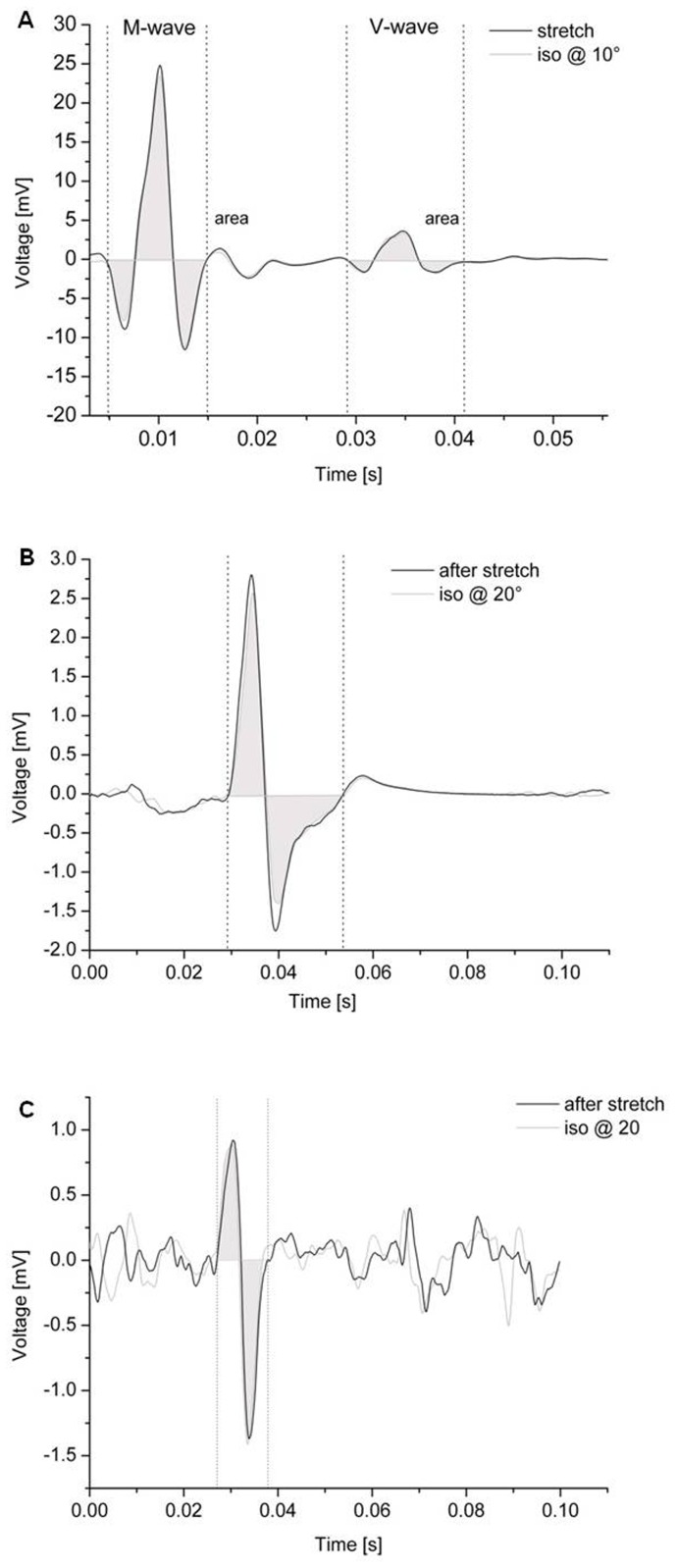
Example data of soleus (SOL) muscle responses to different stimulation types. A (top) M-waves and V-waves elicited by electrical motor nerve stimulation (ENS) during isometric contractions at 10° and during stretch. B (middle) MEPs and C (bottom) shows CMEPs elicited by TMS and cervicomedullary stimulation (CMS), respectively, both during isometric contractions at 20° and after stretch. The size of motor responses was determined by areas under the curves between cursors which were set at the initial and final deflection from baseline. Note the different time scale of figure A.

For the determination of FE and RFE, plantar flexion torque was measured at the time of stimulation 1 and as the mean torque over a 500 ms window prior stimulation 2. Torque of the lengthening contractions was normalised to the corresponding torque of the isometric reference contractions. Similarly, pre-stimulus EMG activity of soleus, medial gastrocnemius and tibialis anterior muscles for isometric and lengthening contractions was calculated as the root mean square amplitude over a 50 ms window prior to stimulation 1 and over a 500 ms window prior to stimulation 2 (Fig. 2).

Areas of M_max_, V-waves, CMEPs and MEPs were calculated from the initial deflection from EMG baseline, which corresponds to the latency of evoked response, to the last crossing of the horizontal axis [38]. The time between the onset and last crossing of the response gave the duration of the evoked response (Fig. 2). For each subject the mean area of the evoked response was computed from all single trials within each contraction condition and within each stimulation type. V-waves, CMEPs and MEPs were normalised to their corresponding M_max_ and all parameters from the lengthening contractions were normalised to the isometric reference contractions.

### Ultrasound Measurements

After data had been collected for all 10 subjects, 3 subjects were invited back to repeat the experiment while ultrasound was recorded to measure MG fascicle length changes. This was done to verify that the muscle fascicles are actually lengthening during the type of eccentric contraction performed in the experiment. Although results focus on soleus, medial gastrocnemius muscle was chosen for ultrasound measurements due to space limitations caused by EMG placement on soleus muscle and due to expected quality of the ultrasound pictures. However, Kawakami et al. [39] showed similar changes in fascicle length for medial gastrocnemius and soleus muscles during contractions. A PC based ultrasound system with a flat shaped 96-element probe (LV7.5/60/96, B-mode, 6 MHz, 65 mm depth; Telemed, Vilnius, Lithuania,) was used to image medial gastrocnemius muscle fascicles at a sampling frequency of 18 Hz. The probe was placed over the belly of medial gastrocnemius and the location of the probe on the skin was marked with indelible marker for consistent placement of the probe throughout the experiment. The probe was then secured using a compressive bandage to minimise movement relative to the skin. Data analysis was performed offline using automated ultrasound tracking algorithms [40]. Due to the limited number of subjects tested with ultrasound, statistics were not calculated but descriptions of fascicle behaviour reported.

### Statistics

All data was tested for normality by using the Kolmogorov-Smirnov test. A two-way repeated measures ANOVA (factor1 “contraction type”, factor2 “stimulation type”) and Bonferroni post-hoc comparisons served to test for differences in absolute torque between isometric and lengthening contractions, as well as between contractions with different superimposed stimulations. A further one-way repeated measures ANOVA (factor “stimulation type”) using lengthening torque normalised to the isometric reference served to test if the amount of FE and RFE (in %) differed between stimulation techniques. Pre-stimulus EMG activity of the analysed muscles was pooled for all stimulation techniques and paired Student’s t-tests were used to check for differences between isometric and lengthening contractions. Differences in the size of muscle responses (M_max_, V-waves, CMEPs and MEPs) between contraction types were tested at time instances by paired Student t-tests. A further paired Student t-test was used to find potential differences between the relative sizes of muscle responses normalised during and following lengthening. Significant differences were established when *p≤*0.05. Values in Results are presented as mean ± standard deviation.

## Results

### Plantar Flexion Torque, Fascicle Behaviour during Lengthening, and Muscle Activity

**Figure 3:**
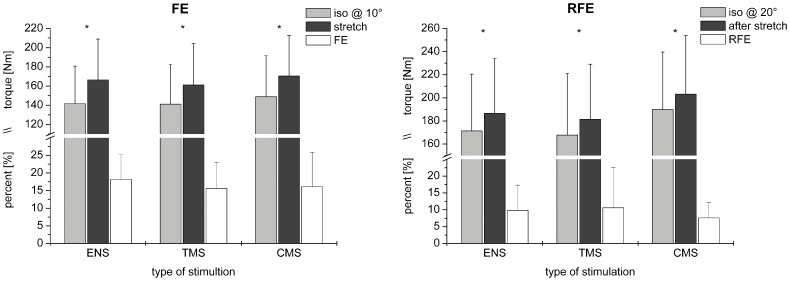
Joint torque for isometric contractions at 10° and 20° (light grey) and stretch contractions during and after stretch (dark grey, left and right, respectively). Asterisks indicate significance (p≤0.05) between purely isometric torques and torques produced during and after the lengthening contractions, respectively. White columns show FE during stretch (left) and RFE after stretch (right). Torque, FE and RFE are shown for electrical motor nerve stimulation (ENS), TMS and cervicomedullary stimulation (CMS). Note the interception of the y-axis with the lower part representing the percentage of force enhancement and the upper part representing absolute torque values (Newton meter).

**Figure 4:**
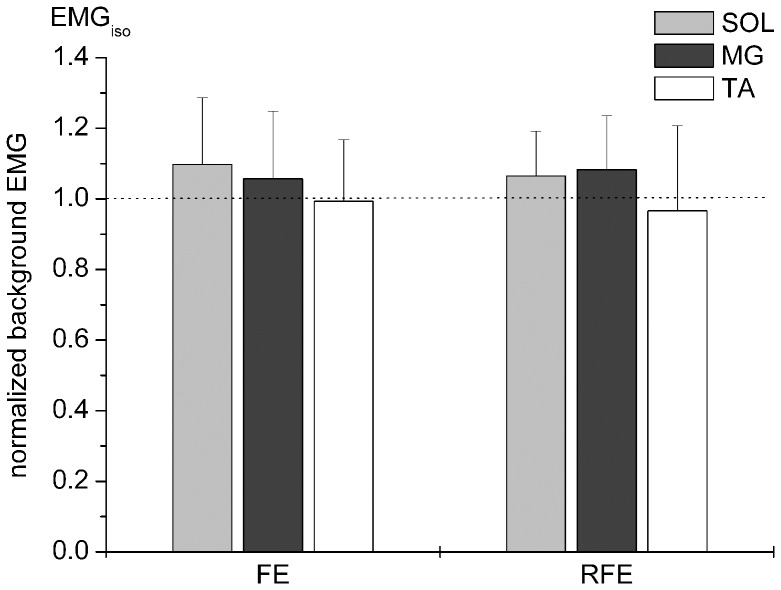
Level of pre-stimulus EMG activity of soleus (SOL), medial gastrocnemius (MG) and tibialis anterior (TA) muscles during and after the lengthening contractions normalised to the purely isometric reference MVCs. Data was pooled for all subjects and stimulation techniques. The dotted horizontal line indicates the normalised isometric reference size 1.

The muscle stretch applied by the 20° movement of the dynamometer resulted in an average medial gastrocnemius fascicle lengthening of 4.9±1.7 mm (n = 3). During the lengthening contractions, plantar flexion torque (n = 10) always exceeded the corresponding torque produced during the isometric reference contractions (F_(1;9)_ = 141.76, p≤0.05) and did not differ between stimulation techniques (F_(2;18)_ = 1.952, p = 0.171). A mean difference in torque of 22.2±8.3 Nm between lengthening and isometric conditions resulted in an average FE of 16.8±7.8% for contractions with superimposed motor nerve stimulation, TMS and cervicomedullary stimulation (Fig. 3). Following lengthening (2.5 - 3 s after the completion of the stretch) plantar flexion torque was significantly greater than isometric T_pf_ (F_(1;8)_ = 28.26, p≤0.05). Although plantar flexion torque during isometric and eccentric contractions with superimposed cervicomedullary stimulation exceeded torque of isometric and eccentric contractions with superimposed motor nerve stimulation and TMS (F_(2;16)_ = 9.242, p≤0.05), RFE did not differ in comparison between stimulation techniques (F_(2;16)_ = 0.973, p = 0.399). The increase in plantar flexion torque of 14.0±8.9 Nm after lengthening compared to isometric resulted in an average RFE of 9.3±8.3% for contractions with superimposed stimulations (Fig. 3). Furthermore, at both instances in time (stimulation 1 and stimulation 2), pre-stimulus EMG of soleus, medial gastrocnemius and tibialis anterior muscles was similar compared between the isometric and lengthening contractions (p>0.05) (Fig. 4).

### Evoked Muscle Responses during and Following Lengthening

**Figure 5:**
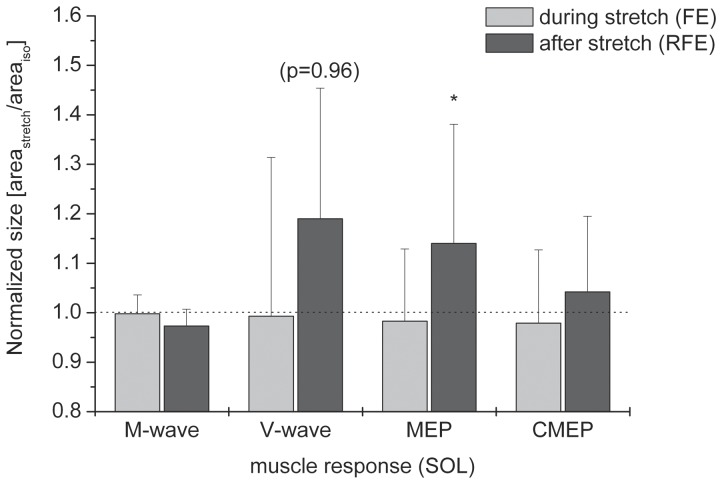
Size of soleus muscle responses to different superimposed stimulations during stretch (light grey) and after stretch (dark grey) normalised to the isometric reference contractions. V-waves, MEPs and CMEPs are further normalised to the sizes (areas) of their corresponding M-waves. The asterisk indicates significance (p≤0.05) when comparing the sizes of responses during and after lengthening to the isometric references. The dotted horizontal line indicates the normalised isometric reference size.

At the time of stimulation 1 mean M_max_ and V-wave areas, elicited by motor nerve stimulation, did not differ between lengthening and isometric MVCs (p = 0.844 and p = 0.7, respectively). Average relative sizes of M_max_ and V-waves normalised to the isometric reference were 1.0±0.04 and 0.96±0.23, respectively. Mean MEP and CMEP areas were not significantly different between lengthening and isometric MVCs (p = 0.896 and p = 0.323, respectively). Normalised sizes of MEPs and CMEPs were 0.98±0.16 for both responses (Fig. 5).

At stimulation 2, 3 s after stretch, no differences in area were found for M_max_ and CMEPs when compared between isometric and eccentric contractions (p = 0.127 and p = 0.356, respectively). Mean V-wave areas following lengthening exceeded those during isometric contractions but did not show significance (p = 0.096), whereas MEP areas were significantly larger (p≤0.05) after stretch compared to isometric. Mean M_max_, V-wave, CMEP and MEP areas normalised to the responses obtained during the purely isometric reference MVCs were 0.97±0.03, 1.19±0.26, 1.14±0.024 and 1.04±0.15, respectively (Fig. 5). Comparing the relative sizes of the muscle responses during and following lengthening showed MEPs and V-wave areas to be significantly larger (p≤0.05) after stretch than during stretch while no differences for M_max_ (p = 0.208) and CMEP areas (p = 0.483) were observed. Absolute sizes of all evoked responses expressed as percentages of M_max_ are presented in Table 1.

**Table 1:** V-waves, MEPs and CMEPs for the different contractions conditions.

	muscle response in soleus		
contraction condition	V-wave [% *M_max_*]	MEP [% *M_max_*]	CMEP [% *M_max_*]
iso @ 10°	16.0±10.0	52.7±40.4	39.6±13.1
FE	16.5±10.8	52.4±41.8	37.7±9.5
iso @ 20°	13.3±7.2	50.3±41.5	37.8±12.4
RFE	15.4±8.2	55.3±44.4	40.1±17.1

Size of soleus muscle responses are expressed as percentage of the corresponding M-wave (*M_max_*). Values are means ± standard deviation.

## Discussion

This study was designed to investigate neural excitability during and after maximal voluntary lengthening contractions. Therefore, the areas of MEPs, CMEPs and V-waves as observed during and after lengthening were compared to those of purely isometric reference contractions at corresponding muscle lengths. The main finding was that the sizes of MEPs, CMEPs and V-waves did not differ between lengthening and isometric MVCs. However, during the sustained contractions after stretch MEPs were larger than during purely isometric contractions and although not showing significant differences, there further was a trend towards larger V-waves after lengthening compared to purely isometric MVCs.

### Eccentric Torque Production during Lengthening MVCs

In contrast to previous findings on elbow flexor and knee extensor muscles [5], [7], [13], our results show the torque produced during a lengthening contraction is not limited to that which is produced isometrically at comparable muscle length. We further did not observe pre-stimulus EMG and/or muscle activation during lengthening to be lower than during isometric contractions [6], [10]–[12]. On the other hand, our results are in line with a number of studies that also showed eccentric torques of adductor pollicis, quadriceps femoris and tibialis anterior muscles to exceed the isometric maximum by a factor 1.1 to 1.5 at a similar level of EMG during lengthening compared to isometric [14], [15], [17]. That is during lengthening more torque is produced by the same motor output as observed during isometric MVCs. Due to contradictory literature it therefore remains uncertain as to whether the previously observed absence of enhanced torques during maximum voluntary lengthening contractions is only due to neural inhibition [9] via a reduction in motor output as a consequence of afferent input associated with stretch, or additionally due to the subjects’ inability to fully activate their muscles via incomplete voluntary activation (VA).

The assumption of incomplete VA is supported by the torque-time trace of the isometric reference contraction shown by Duclay et al. [23] in their Figure 1, as after stimulation there is an additional superimposed twitch torque in response to stimulation of the voluntarily activated plantar flexor muscles. The appearance of such a twitch implies that voluntary activation was incomplete, even during the purely isometric contraction where no ‘lengthening’ inhibition would be expected to occur. To ensure highest possible activation during stretch, eccentric contractions should be preceded by a maximal isometric contraction [41], [42]. In several studies where subjects failed to produce eccentric torques higher than their isometric maximum, lengthening contractions either started from low pre activation levels [6], [7], [11], [13] or were performed as combined shortening-stretch cycles [12] although it is well known that force production following shortening is depressed [43]. Additionally, training status of subjects seems to influence the ability of generating enhanced eccentric torques [44].

Accordingly, recent studies on plantar and elbow flexors [20], [21], [23] attempted to achieve maximal pre-activation of the muscles by instructing their subjects to perform maximum isometric contractions before stretch. However, their subjects still failed to produce higher eccentric torques during stretch, which could be entirely due to inhibition at the neural level or alternatively that during the isometric pre activation their subjects were not able to fully activate their muscles. In contrast, mean level (pooled) of VA of our subjects for all isometric contractions at 10° dorsiflexion was 94.4±5.9%. If only the best trial from each subject was taken into account, mean VA was 99.0±1.7%, with 7 out of 10 subjects being able to maximally drive their soleus muscle. To ensure maximal activation, it is important that there is sufficient familiarization of subjects, but according to the methods sections of the papers under discussion [20], [21], [23] no time was spent on practicing the experimental task and/or maximum voluntary contractions. The impact of such a familiarization is unlikely to elicit structural adaptations as observed after systematic training, but promote positive neuromuscular adaptation through learning [45]. Using this approach we have been able to achieve significant increases in eccentric torque of 17±8% relative to isometric torque at the same joint angle, although contractions were performed on the ascending limb of the force-length relationship. However, it still remains unclear why voluntary eccentric torques are still less than those predicted, or produced, when muscles are electrically stimulated [4], [12], [13].

### Modulation of Neural Excitability during Lengthening MVCs

None of the muscle responses (M-wave, V-wave, MEP, and CMEP) analysed in this study showed any modulation during lengthening compared to purely isometric contractions. With MEPs reflecting properties of neurons at the motor cortex and properties of the spinal motoneuron pool combined with CMEPs relating only to excitability of spinal motoneurons, our results indicate cortical and spinal responsiveness to be similar to that in isometric conditions. This suggests that the major voluntary motor pathways are not subject to substantial inhibition during stretch, which is also consistent with unchanged V-waves during lengthening. Thus our results are insofar in accordance with previous findings that eccentric torque production is likely not limited by voluntary descending drive from the motor cortex [21].

However, in contrast to our findings, it was also argued that cortical output is likely to be enhanced during lengthening MVCs to counteract spinal inhibition. This was concluded from unchanged V-waves in combination with reduced H-reflex amplitudes [20], from increased MEP-to-CMEP ratios when both, MEPs and CMEPs sizes were reduced during lengthening of the elbow flexors [21], and from shorter silent periods after smaller MEPs during lengthening than during isometric MVCs of soleus muscle [23]. The exact spinal mechanism(s) eliciting the assumed neural inhibition during lengthening MVCs still remain elusive, but afferent Ib feedback from Golgi tendon organs, pain receptors, Renshaw cells, reciprocal inhibition by antagonistic co-activation as well as pre- and postsynaptic inhibition of Ia afferents have been suggested [6], [7], [13], [20], [21], [23], [44], [46], [47].

A common explanation for the absence of enhanced torques during lengthening MVCs is a potent tension and activation limiting mechanism to prevent musculo-tendinous tissues from injury [4], [6], [7], [13], which is thought to be related to inhibitory afferent Ib feedback [13], [20], [21], [47]. However, Duchateau & Enoka [19] question a tension-related inhibitory mechanism. Given the tension sensitivity of Golgi tendon organs but no higher torques during lengthening as in the studies that proposed such a mechanism, an excitation of Ib afferents seems unlikely. On the other hand, when there is enhanced torque during lengthening MVCs [14]–[17] that is less than that when muscles are electrically stimulated [2], the suggested tension-related inhibitory mechanism might contribute to the observed differences in torque or force production between voluntarily and electrically activated muscles. However, such inhibition was not reflected in our results.

Another factor that has been attributed with absent force enhancement and spinal inhibition during lengthening MVCs is co-activation of antagonistic muscles and the associated effect of reciprocal inhibition [6], [44], [47]. In contrast to that but similar to others [21], [23], our results revealed similar EMG of antagonistic tibialis anterior muscle during maximal isometric and lengthening MVCs. Therefore reciprocal inhibition cannot be responsible for reduced spinal excitability [23] and/or lower FE compared to electrically stimulated muscles as observed in the present study.

The final neural mechanism related to spinal inhibition that has been promoted by many studies is presynaptic inhibition [20] caused by greater activity of Ia afferents themselves [48] or by homosynaptic postactivation depression [49]. However, during strong voluntary contractions the latter was assumed to have only minor influence on Ia input [18]. Furthermore, Petersen et al. [18] could not find any specific regulatory control of Ia afferents during lengthening contractions of tibialis anterior and therefore conclude that the motoneuron excitability is set by the cortex. This was also suggested by others for the elbow flexor muscles [21], [46], whereas again, unchanged MEPs during lengthening MVCs in the current study do not support the idea of a modulation at the cortical level. However, since the current study did not investigate presynaptic inhibition by measuring the H-reflex under appropriate conditions, future studies are warranted.

A last point to discuss is the possible influence of a subjects’ potential inability to fully activate their muscles voluntarily on the results described in literature. In the current study unchanged MEPs and CMEPs were observed during lengthening compared to isometric MVCs when there was FE of 17±8%. In contrast to that, the two other studies that compared isometric and lengthening MVCs found smaller MEPs [23] or both, smaller MEPs and CMEPs [21] during lengthening compared to purely isometric but no increase in torque. However, when torque during lengthening equals isometric torque, the force actively produced by the muscle during lengthening is probably less than that produced during isometric MVCs due to the extra contribution of passive forces from series elasticity to the overall torque of the muscle. According to the finding that for a given submaximal stimulation intensity MEP as well as CMEP size of soleus increases with contraction intensity [36], reduced MEPs and CMEPs as described earlier [21], [23] might at least partly be due to reduced forces actively produced by the muscles. In terms of the investigation of neural activation strategies for different muscle action types, lengthening torque data not exceeding the isometric conditions therefore confounds the interpretation of modulated excitabilities.

In contrast to our hypothesis that modulations in neural control depend on feedback via spinal loops, our results demonstrated that the major voluntary motor pathways are not subject to substantial inhibition during lengthening MVCs, if subjects are able to achieve maximal voluntary activation. However, the effects of afferent feedback on neural control caused by enhanced torque and approximate 5 mm fascicle stretch as observed in the current study remain unclear and need further investigation.

### Isometric Torque Production after Lengthening MVCs

The result of 9±8% enhanced torque observed 2.5–3 seconds after an eccentric lengthening contraction is well in line with previous findings on RFE [14], [15], [17], [25], [26], [31]. Further, the current results confirm former observations that enhanced torque production after lengthening compared to purely isometric contractions does not require extra muscle activation at least to what is reflected by pre-stimulus EMG recordings. Therefore, passive structures such as titin [50] remain as candidates to explain higher torques after lengthening. Finally it should be noted that one subject did not show RFE after the lengthening contractions in the current study. This phenomenon of so called non-responders was observed by others [16], [26], [31] but its origin remains unclear.

### Modulation of Neural Excitability during Isometric MVCs Following Lengthening MVCs

During the isometric steady state phase following lengthening MVCs, MEPs and V-waves exceeded those observed during purely isometric MVCs at corresponding muscle length whereas M_max_ and CMEPs remained unchanged. Furthermore, our results revealed larger MEPs and V-waves after compared to during stretch. The increase in MEP size despite stable CMEPs suggests an increase in cortical excitability, and the trend towards increased V-wave amplitude suggests greater motoneuronal output or increased stretch reflex excitability. However, due to the isometric character of the MVCs following stretch the trend towards increased V-waves seem unlikely to be caused by enhanced stretch reflex excitability. Although this illustrates for the first time that neuromotor pathways are altered during the expression of RFE, it is unclear whether these changes underlie the enhanced torque.

Regarding muscle activation levels when there was RFE during maximal and submaximal contractions after lengthening, the results of previous investigations point towards an enhanced effectiveness of the neuromuscular system [32], [33]. Assuming that all motor units have already been recruited during the purely isometric MVCs, the larger MEPs as well as larger V-waves observed after lengthening might support this idea, if the same active MUs produce an enhanced force after lengthening due to the increase in cortical excitability and/or greater motoneuronal output. However, since the enhanced neural drive observed after lengthening MVCs was not reflected in the pre-stimulus EMG recordings, this could also be related to a passive mechanism. Given the calcium sensitivity of the tension produced by the giant protein titin [51], modified release and influx of calcium in the muscle cells due to greater motoneuronal output after the lengthening MVCs might also have contributed to the observed development of RFE. Despite the tentativeness of the neurophysiological mechanisms discussed above, the modulation of MEPs and V-waves after lengthening showed that both, central and passive mechanisms remain as candidate mechanisms underpinning RFE.

In conclusion, unchanged V-waves, MEPs and CMEPs as observed in SOL muscle during maximal voluntary lengthening contractions revealed similar cortical and spinal responsiveness to that in isometric conditions, suggesting that the major voluntary motor pathways are not subject to substantial inhibition during stretch. However, although torque produced during the lengthening MVCs exceeded that which was produced isometrically at a comparable muscle length, the amount of FE was less than that produced, when muscles are electrically stimulated. Due to absent modulations of neural excitability the origin of this difference remains unclear. The results after lengthening revealed RFE accompanied by larger MEPs and a trend to larger V-waves. In combination with unchanged CMEPs this indicates enhanced cortical excitability and greater motoneuronal output or increased stretch reflex excitability. For the first time, this documents a modulation of the neuromotor pathways during the occurrence of RFE, which indicates that the underlying mechanisms of enhanced torque after lengthening contractions are probably not purely mechanical in nature.
